# The State of Shared Decision‐Making in Immune Thrombocytopenia: A Narrative Review and Health Care Provider Perspective

**DOI:** 10.1002/hsr2.72973

**Published:** 2026-08-26

**Authors:** Shivi Jain, Kelsey Kuhel, Rose Cree, Michael D. Tarantino

**Affiliations:** ^1^ Division of Hematology Rush University Medical Center Chicago Illinois USA; ^2^ The Bleeding & Clotting Disorders Institute Peoria Illinois USA; ^3^ University of Illinois College of Medicine‐Peoria Peoria Illinois USA

**Keywords:** autoimmune disease, immune thrombocytopenia, multidisciplinary care team, quality of life

## Abstract

**Background and Aims:**

Advances in the treatment of immune thrombocytopenia (ITP) have broadened the landscape of treatment options for patients. Shared decision‐making, a collaborative process between a patient and their health care team, is encouraged for these patients. The ultimate goal of shared decision‐making is to personalize treatment to achieve outcomes that are most important to the patient. However, there are no standard models for shared decision‐making in ITP. This narrative review provides a general overview of shared decision‐making, with a focus on ITP.

**Methods:**

The PubMed database was searched for studies related to shared decision‐making in general, shared decision‐making in ITP, and shared decision‐making in hemophilia. Gray literature—including reports from professional and patient organizations focused on supporting patients with ITP and hemophilia—was also searched to capture a more comprehensive perspective.

**Results:**

Select shared decision‐making models established in hemophilia, a bleeding disorder with both clinical and non‐clinical similarities to ITP, are discussed for their potential as guides for developing similar models in ITP. Patient characteristics and individual situations that can have an impact on the shared decision‐making process are detailed. Finally, we consider approaches to shared decision‐making in ITP with an emphasis on the experience of the authors as members of the Comprehensive Care team at the Comprehensive ITP Clinic at The Bleeding & Clotting Disorders Institute in Peoria, Illinois, which is the first Comprehensive Care Clinic for patients with ITP in the United States.

**Conclusion:**

The development of shared decision‐making models specific to ITP may improve the consistency at which this approach is utilized. Shared decision‐making should be tailored to each individual patient's specific needs and treatment priorities/concerns. Utilization of ITP‐specific shared decision‐making models is expected to improve overall patient care and enhance the communication between patients and their healthcare providers.

## 1 Introduction

Immune thrombocytopenia (ITP) is an autoimmune disease characterized by a transient or persistent reduction in blood platelet count to < 100 × 10^9^/L [1]. The annualized incidence of ITP in the United States is 6.1 per 100,000 persons (all ages) [2] and 8.8 per 100,000 persons < 18 years [3]. Options for first‐line treatment include intravenous immune globulin, corticosteroids, and anti‐D immune globulin [4, 5]. Second‐line treatments are available for patients who do not respond, who do not have a durable response, or who relapse after achieving a durable response to first‐line therapy. Options with the strongest evidence for clinical benefit are rituximab (anti‐CD20), fostamatinib (spleen tyrosine kinase inhibitor), and thrombopoietin receptor agonists [5]. Azathioprine, cyclosporine, cyclophosphamide, danazol, dapsone, mycophenolate mofetil, and vinca alkaloids are also available as third‐line options; however, the evidence for their efficacy and/or safety is less robust [5]. Splenectomy can also be considered when medical therapies fail [5].

Given that patients with ITP have a variety of options for treatment, it is important to consider the best approach for making treatment decisions. Guidelines published by professional societies offer up‐to‐date expert consensus guidance to health care providers (HCPs) to support evidence‐based treatment decisions [4, 5]. These guidelines provide the foundation for treatment recommendations. Traditionally, many physicians have practiced a dogmatic approach to patient treatment decisions; however, more recently, shared decision‐making has gained popularity [6]. This model promotes patient involvement in treatment decisions to achieve patient‐centered outcomes. In this collaborative process, HCPs support patients to reach a decision about a specific course of action related to their health care [7]. Ultimately, conversations around treatment decisions should bring the HCPs' expertise together with the preferences, social circumstances, personal circumstances, beliefs, values, and goals of the patient [7]. During this process, HCPs should ensure patients understand the benefits, risks, and possible consequences of each treatment option [8].

When considering treatment decisions in ITP specifically, the most recent ITP treatment guidelines from the American Society of Hematology encourage a focus on patient education and shared decision‐making, particularly regarding second‐line therapy [4]. Ghanima et al. developed an algorithm for the selection of second‐line therapy in adults with ITP that encourages an individualized treatment plan while considering patient values and preferences [9]. Additionally, the establishment of research models to assess, support, and understand patient values and preferences in shared decision‐making was identified as a high research priority by the American Society of Hematology [4]. The updated international consensus report on the management of primary ITP emphasized that treatment goals should be individualized to both the patient and the phase of ITP and that the patient's quality of life is an important consideration when deciding on treatment options [5]. Further, it was stressed that patients and their families should be included more in treatment decision‐making [5].

Hemophilia is a bleeding disorder that shares many clinical and non‐clinical aspects with ITP (Table 1) [4, 5, 10, 11, 12, 13, 14, 15, 16, 17, 18, 19]. Comprehensive Care for people with hemophilia is long established and includes national networks of Hemophilia Treatment Centers [13]. At these specialized centers, patients receive focused education and training about bleeding disorders [20]. Additionally, a major focus of care is on preventive medicine. Patients in this model have access to multidisciplinary expertise and care and receive individually tailored management plans [20, 21]. This approach has been quite successful. This Comprehensive Care model has been applied to the care of patients with ITP at The Bleeding & Clotting Disorders Institute (BCDI) in Peoria, Illinois, formally since August, 2017 [10]. Patients utilizing this clinic have reported an overall improvement in the ease with which they can perform their activities of daily living.

**TABLE 1:** Comparisons between hemophilia and immune thrombocytopenia.

	Hemophilia	Immune thrombocytopenia
Epidemiology [10]	Inherited genetic disorder	Acquired autoimmune disorder
Disease course [10]	Bleeding disorder Affected starting at birth Lifelong duration Main bleeding sites: – Joints – Mucocutaneous – Gastrointestinal – Genitourinary	Bleeding disorder Affected at any age Lifelong duration (from time of diagnosis) Main bleeding sites: – Mucocutaneous – Gastrointestinal – Genitourinary
Treatment dynamics	Prophylaxis with FVIII concentrates or non factor therapies [11]	First‐line corticosteroids, IVIG, or anti‐D Ig [4, 5] Second‐line thrombopoietin receptor agonist, rituximab, or fostamatinib [4, 5]
Non‐clinical aspects	Costly treatment [12] National network of comprehensive care centers [13]	Costly treatment [14, 15] Lack of comprehensive care centers
Health‐related quality of life	Pain [16] Anxiety, depression [16] Work/school absenteeism [17] Social functioning [18]	Fatigue [19] Reduced energy levels and capacity to exercise [19] Limits ability to perform daily tasks [19] Impact of emotional well‐being [19] Reduced work hours [19] Decreased work productivity [19]

Abbreviations: Ig, immunoglobulin; IVIG, intravenous immunoglobulin.

This narrative review discusses the role of shared decision‐making, with a focus on ITP. We review select shared decision‐making models established in hemophilia as a potential guide to shared decision‐making in ITP. Additionally, patient characteristics and individual situations that may impact the shared decision‐making process are discussed. Finally, the approach of HCPs to shared decision‐making, including the approach based on author experience, is discussed. The content was developed by searching the PubMed database for studies related to shared decision‐making in general, shared decision‐making in ITP, and shared decision‐making in hemophilia. Furthermore, a search of gray literature—including reports from professional and patient organizations focused on supporting patients with ITP and hemophilia—was conducted to capture a more comprehensive perspective. This narrative review did not require ethical approval or informed consent, as no patients were involved.

## 2 Current Use of Shared Decision‐Making and Evidence Supporting Its Impact on Treatment Decisions and Patient Outcomes

General benefits of shared decision‐making include greater overall satisfaction of both patients and clinicians, better outcomes, greater satisfaction with care, better alignment of treatment goals with patient values, and better patient–physician interpersonal connections. Patients who feel more knowledgeable about their condition have a greater satisfaction with their treatment, and clinicians feel satisfied about providing high‐quality care supported by their patients [22, 23]. Patient anxiety is reduced, and adherence to medication prescriptions is improved [22, 24]. This, in turn, reduces clinician burnout [22]. Increased patient engagement also improves staff morale and motivation [25]. With shared decision‐making, patients' concerns are acknowledged and their questions are answered, ensuring that they can respect and own treatment decisions [22]. It has also been shown to improve patient outcomes [24, 26].

Systems and decision aid tools facilitate effective shared decision‐making [27]. For example, patients with oncologic disorders often have multiple treatment options without a clear best choice. Shared decision‐making studies with oncology patients have shown that decision‐making aids improve engagement, facilitating decisions better aligned with their values and recent scientific evidence [28]. Such aids should incorporate interactive, multi‐directional communication between clinicians, caregivers, and patients with consideration for the diversity of ethnic, sociocultural, and racial backgrounds and preferences [29, 30]. Current limitations to better implementation of shared decision‐making include the lack of data linking shared decision‐making to better outcomes, complexity of decision‐making in the absence of comparative trials, the need for high‐quality decision aids, lack of specialized clinician training, and the need to tailor the decision encounter based on the social, environmental, and cultural perspective of the patient [27, 30, 31].

Personal health literacy is a complex factor that greatly impacts shared decision‐making. A patient's ability to find, understand, and utilize health information about their condition is at the core of informed consent and health care decision‐making [32]. Results from the 2003 National Assessment of Health Literacy show that 22% and 14% of adults had basic or below basic health literacy, respectively [33], a concerning statistic when viewed from the lens of rare or complex disease states, such as ITP. Discussions around shared decision‐making must acknowledge that some patients have limitations in their ability to understand their own health information, limiting their ability to meaningfully contribute to their own health care decisions. At the same time, providers and their organizations must make genuine efforts to provide health care information in an equitable way, accounting for different preferred learning styles, primary languages, and varying levels of literacy. Ultimately, a physician must be aware of their patient's ability to understand the health information that is communicated and be an equal shareholder in the responsibility for providing health information that is understood and can be utilized to make informed health choices.

So, where do we stand with the use of shared decision‐making in the treatment of ITP? To answer this question, it is important to understand recent patient perspectives on issues related to shared decision‐making. The ITP Support Association Patient Perception Survey (2020) reported that 35% of patients did not have or were not aware of their doctor having a treatment plan for them, indicating that over one‐third of patients may have not discussed or did not remember treatment decisions with their HCP [34]. Patients noted that they were more likely to feel empowered if they were able to discuss treatment and care options with an ITP clinical nurse specialist; however, 75% of patients surveyed did not have access to or were not aware that they had access to an ITP clinical nurse specialist [34]. Additionally, 26% of patients reported they had not been told about possible treatment side effects, and some patients had been put on a specific treatment against their preference [34]. A review on unmet needs from the perspective of patients with ITP revealed that they wished to be provided with standardized protocols that could be shared with their family members, workplaces, and schools that provide guidance on how to manage bleeding events [35]. Patients also wanted to be involved in their own management plan in partnership with their HCP and had a desire to be educated on all available treatment options, regardless of insurance coverage [35]. Parents of children with refractory ITP had mixed experiences with shared decision‐making; some reported that they were never given more than one treatment option, while others reported a back‐and‐forth conversation with their physician covering available treatment options [36]. Clearly, patients and caregivers have opinions on how treatment should be implemented, as they noted that quality of life, treatment effectiveness, comfort, and medication characteristics, such as side effects, were important factors impacting decision‐making [36]. Another report indicated a lack of shared decision‐making at the time of initial diagnosis of pediatric ITP and found that families experienced an extreme emotional toll upon diagnosis, which should be accounted for when considering implementation of shared decision‐making [37]. These reports point to a deficiency in shared decision‐making in ITP for some patients. To help address this deficiency and improve shared decision‐making in ITP, the ITP Support Association has put together a shared decision‐making toolkit for patients, which helps guide them through this process [38].

## 3 Shared Decision‐Making in Hemophilia as a Model for ITP

For people with hemophilia, shared decision‐making is particularly important for deciding on a plan for prophylactic treatment and, more recently, for deciding whether gene therapy is an appropriate option. Much effort has been made in the hemophilia community to address optimal approaches toward shared decision‐making. Learnings in this field may be applicable to developing shared decision‐making models in ITP. A key factor for shared decision‐making is access to a multidisciplinary team. With the established use of Hemophilia Treatment Centers, people with hemophilia have access to multidisciplinary medical teams who can contribute to treatment decisions and personalized treatment plans (examples of Comprehensive Care team member roles are shown in Table 2) [10, 39, 40, 41]. A lack of such Comprehensive Care centers for people with ITP may contribute to a lack of standard shared decision‐making practices for these patients.

**TABLE 2:** Roles for Comprehensive Care team members in shared decision‐making.

Comprehensive Care team member	Role in shared decision‐making
Hemophilia [31, 32, 33]	
Hematologist	Medical director Address acute manifestations of hemophilia
Nurse coordinator	Coordinate provision of care Educate patients and their families
Nurse practitioner	Mental health care Assisting team members with treatment as needed
Pharmacist	Provision of information on therapeutic drugs to relevant team members Management of drug inventory Point of contact for patients Patient education Streamline access to financial assistance programs
Physical therapist	Joint evaluation and treatment Educate patients regarding non‐hematologic strategies for addressing bleeding episodes Address exercise requirements and potential
Medical social worker	Mental health care Supporting social welfare
Dentist	Provide effective dental care tailored to the unique needs of people with hemophilia Instruct patients on proper brushing techniques Provide education on effective preventative dental care methods
Specialized coagulation laboratory	Provide access to accurate and precise clotting factor assays and inhibitor testing
Immune thrombocytopenia	
Hematologist	Discussion of medication experiences Presentation of alternative choices Setting goals for the near and far future
Nurse coordinator	Thorough discussion of available treatment options
Nurse practitioner	Provide treatment education handouts Discuss adverse effects, administration routes/logistics, and restrictions related to specific treatment plans Understand and consider patient concerns and personal obstacles Aid the patient in choosing the most appropriate treatment plan Help the patient understand which treatment goals are realistic or attainable
Pharmacist	Provision of information on therapeutic drugs to relevant team members Management of drug inventory Point of contact for patients Patient education Streamline access to financial assistance programs
Physical therapist	Musculoskeletal evaluation Injury prevention through overall fitness, core strength Gait assessments and treatment Participates in global wellness program for patients with ITP
Medical social worker	Determination of how socio‐medical, socio‐cultural, and socio‐economic issues may influence treatment success: o Medication adherence o Employment o Aspects of quality of life
Dentist	Screen for dental diseases that may lead to invasive procedures Counseling on dental disease prevention
Immunologist	Evaluation of comorbid conditions and underlying immune system disorders that may have an impact on the patient's quality of life and/or treatment success
Registered dietician	Addresses obesity and related comorbidities

A British Columbia Hemophilia Adult Team focus group identified three phases of the health care team/patient partnership journey that can be used to continuously develop an individualized prophylaxis regimen. The three phases are engagement, in which the patient prepares for their first clinic visit; assessment, a patient‐driven process of assessment by a multidisciplinary care team; and individualization, which involves follow‐up and decision‐making [42]. A 12‐month cohort study reported a decreased annual bleed rate and improved quality of life when adopting this strategy to align the prophylaxis treatment plan with patient priorities [43, 44].

Another, more recent approach to shared decision‐making is the hemophilia‐specific, patient‐centered outcome measure, GOAL‐Hēm [45]. This measure allows people with hemophilia to set their own individualized goals for treatment and subsequently monitor them [45]. It was developed through a combination of clinician workshops aimed toward developing a hemophilia‐specific goal menu and the collection of qualitative data from patients and their caregivers, which allowed for fine‐tuning of the goal menu [45]. A key component of the GOAL‐Hēm is the use of goal attainment scaling, which allows both patients and clinicians to track progress toward meaningful treatment goals [45]. Following initial development, the model was validated in a 12‐week feasibility study that set goals in three domains: managing hemophilia, impact on life, and hemophilia complications [45]. Patients with hemophilia addressed a prespecified goal or selected a goal in collaboration with their physician and then created an individualized 5‐point scale to measure change [45]. Users reported that focusing on what was important to them led to the feeling that they had greater control over their disease management [45]. The study reported large effects in patient‐ and clinician‐rated goal attainments that were considered clinically meaningful [45]. Following the feasibility study, goals and descriptors were further refined to provide a more straightforward menu [45]. An advantage of GOAL‐Hēm is its sensitivity to small, meaningful changes; however, successful individual development and implementation of the model requires significant input from both people with hemophilia and HCPs. The development journey of GOAL‐Hēm may be a useful road map for the development of other patient‐centered outcome measures that involve shared decision‐making.

Gene therapy has recently been approved for treatment of hemophilia A and B. In anticipation of this, the Council of Hemophilia Community hosted a series of roundtable meetings with input from patient groups. From this, they defined the patient journey and created a shared decision‐making resource to cover questions and concerns for patient/HCP discussion through each of the five stages of gene therapy (information seeking, decision‐making, treatment initiation, and short‐ and long‐term post‐gene therapy follow‐up) [46]. In creating this tool, the committee noted that focusing on the importance of physician–patient communication is key to any shared decision‐making model, regardless of the indication. Communication can be improved by avoiding unnecessary detail and jargon, avoiding assumptions of high or low patient health literacy, preparing written materials with only key points and graphics/visuals, and utilizing the “teach‐back” method where the patient reiterates what they have learned back to the physician in their own words [46]. Discussion with members of the multidisciplinary health care team other than the physician is also important. Team members should be prepared to engage in conversation around patient education and decision‐making, as this can help ease the time burden on physician visits and lend consistency to the information received by the patient [46].

## 4 Aspects That Affect Shared Decision‐Making on an Individual Patient Level

Many individual patient factors can impact shared decision‐making. Social determinants of health are a set of non‐medical factors, such as the conditions in which a person is born, grows, learns, lives, and works, that influence health outcomes [47]. Specific examples include access to health care, race, ethnicity, socioeconomic status, education, employment, neighborhood and physical environment, housing, education, and social support networks [48, 49]. Barriers to effective shared decision‐making for immigrant patients include difference in language background, different values about health and illness, differences in role expectations, and provider prejudice [50]. These factors affect health and health equity and may affect patient experience with shared decision‐making [48, 49].

A survey of patients with hemophilia found that economic status, education level, and age significantly influenced whether a patient was willing to participate in shared decision‐making [51]. At the patient level, poor quality patient–clinician communication is associated with having limited English language proficiency, being a member of a racial/ethnic minority, and having low health and digital literacy [51]. Other factors influencing effective participation in shared decision‐making include employment status, rigors of the job, transportation access, insurance coverage, disposable income, life obligations, cultural norms, and views on the medical establishment or prescription medication. These factors must be considered in the context the shared decision‐making approach. Tailoring conversations within the context of each patient's environmental, social, and cultural situation is expected to make the shared decision‐making process more acceptable, effective, and inclusive to patients from diverse backgrounds [30].

Individual patient treatment goals and concerns are also an important consideration. Patients with a desire to become pregnant or who are pregnant will likely have different treatment goals and priorities related to medication preference. For adolescents with ITP, it is important to consider treatment approaches that allow the patient to participate in activities and normal life as much as possible, and the effects of steroid treatment, for example, mood changes, sleep disturbance, and weight gain, should be clearly communicated [38]. A small survey of people with hemophilia and mothers of boys with hemophilia found that participants considered ease of use (e.g., easier handling, fewer injections, and alternative administration), the added value of new treatments, concern over reimbursement for long‐term treatments, and concern for coverage of medication usage due to high‐risk activities as factors for treatment decision‐making [52].

Finally, relevant comorbid health conditions should also be factored into individualized shared decision‐making [53]. Findings from focus groups with older patients (≥ 65 years) with multiple comorbidities and general practitioners revealed a need for greater awareness of multimorbidity guidelines and that shared decision‐making training for physicians should include education on how to communicate clinical uncertainty [54].

## 5 HCP Strategies for Shared Decision‐Making: How Does Approach Play Into Patient Perception?

A physician's decision‐making approach can impact patient participation in shared decision‐making. A measured/rational decision‐making style has been associated with better patient participation in shared decision‐making than hastened/intuitive decision‐making [55]. Consideration of patient preferences regarding the sharing of information is also important. Factors associated with poor quality patient–clinician communication on the clinician level include a lack of communication skills to successfully engage in shared decision‐making, being less culturally competent, and the presence of unconscious biases [51].

Optimal management of patients with bleeding disorders includes early detection, preventive measures, treatment, educational and psychosocial support, and patient advocacy. Team members at the Comprehensive ITP Clinic at BCDI include a hematologist, nurse coordinator, nurse practitioner, pharmacist, physical therapist, medical social worker, dentist, immunologist, and dietician [10]. At the Comprehensive ITP Clinic, every patient visit includes discussions with each specialist to provide an integrated approach to care. Specifically, registered nurses (RNs), advanced practice nurses, and the hematologist discuss medication experiences, alternative choices, and goals for the near and far future. The immunologist looks for comorbid conditions that may impact quality of life and/or treatment success. The medical social worker covers socio‐medical, socio‐cultural, and socio‐economic issues and how these may influence treatment success, including the impact on medication adherence, employment, and other aspects of quality of life. The registered dietitian discusses other aspects of wellness, including obesity and related comorbidities. The dentist screens for dental disease that may lead to invasive procedures and provides counseling on dental disease prevention. In the interim between visits, the RNs, advanced practice nurse, medical social worker, and hematologist have numerous contacts with patients (averaging 30/year) to stay abreast of health changes, needed procedures, changes in employment or other aspects of financial status, transition to adulthood, and other major life changes. Examples of the roles played by each member of the Comprehensive Care team are listed in Table 2.

## 6 Author Experience: Key Communication Points and Perspectives on Shared Decision‐Making

Shared decision‐making should be incorporated for most, if not all, decisions regarding treatment of ITP, regardless of the phase of disease. Key clinical decision points include the decision to treat, choice of first‐, second‐, and later‐line therapies, treatment modifications during pregnancy or prior to surgery, and the potential for switching medications due to issues with tolerability, lack of efficacy, convenience, or other reasons [4, 5]. When considering first‐line treatment, patients/parents of children should be involved in deciding whether to treat or observe and, if drug treatment is decided upon, whether to use corticosteroids or intravenous immune globulin/anti‐D immune globulin. For second‐line treatment, shared decision‐making should involve a discussion around whether a thrombopoietin receptor agonist or immunosuppressive drug should be used, and if/when splenectomy should be considered.

With regard to the efficacy of treatment, it is important to discuss with patients how quickly they can expect a response with different treatment options. Other important points to discuss with patients include the durability of response, dosing frequency, tablet size, and tablet strength. Patients should also be informed of the convenience/inconvenience of treatment options, such as whether treatment is administered orally or injected, the required frequency of office visits (e.g., in‐office administration requirement), dietary restrictions with certain treatments, and whether dose adjustments are needed based on concomitant medications. Finally, it is important to communicate the known toxicities and adverse effects of each treatment option and to understand the patient's perspective on which potential side effects are acceptable and which are not.

From the hematologist's perspective, the health care team should ensure that the profile and burden of each medication choice are clear and understood by the patient. As noted earlier, health literacy is vitally important to this process. The importance of medication and monitoring adherence should also be emphasized and clearly understood by the patient. Keeping an open line of communication with key caregivers is crucial for sharing information related to changes in health, injuries, anticipated injuries, and anticipated vacations. Finally, it is essential to develop a relationship of trust between the ITP experts and the patient and to include both the patient and their family as part of the Comprehensive Care team.

In geographic areas lacking access to a Comprehensive Treatment Center for ITP, the responsibility to coordinate care for patients with ITP usually falls on the treating hematologist. It is essential that these physicians have the education and expertise necessary to effectively communicate with patients regarding their treatment goals and intended outcomes, as well as the expected adverse effects of each treatment modality. To this end, it is important to empower HCPs with tools to communicate this information adequately. Patients should also be provided with tools to effectively assess the impact of ITP on their daily life, manage their medications and blood counts, and have a clear understanding of treatment goals. Such tools would help resolve discrepancies related to treatment goals between the patient and HCP.

From the viewpoint of nurse coordinators, a shared decision‐making approach should include taking the time necessary to go over available treatment options during a clinic appointment or over the phone. Nurses should provide treatment education handouts for specific treatment options and discuss adverse effects, administration routes and other logistics (i.e., injection vs. oral or home vs. office administration), and restrictions related to specific treatment plans (i.e., diet restrictions). It is also important to listen to patient concerns and their personal obstacles to help them choose the most appropriate treatment plan.

During the course of shared decision‐making, differences may emerge between the treatment goals of the HCP and those of the patient. The approach to resolving such differences requires consideration of many aspects of the patient's life, including organized or casual physical activity. The question of sports participation should also involve shared decision‐making, as this may impact treatment choice and the frequency of platelet monitoring. From the nurse's perspective, differences between the treatment goals of the HCP and patient can be addressed by educating the patient on the goals of their treatment plan and helping them understand which goals are considered realistic or attainable. A discussion of risk vs. benefit from the HCP standpoint will help patients and their families understand the importance of the recommendations. Within reason, HCPs can consider deviating from standard protocols to make the patient's goals more attainable (i.e., less frequent lab draws and/or clinic appointments, or monitoring more frequently to ensure a patient is safe to continue participation in sports). Open discussion between the HCP and patient should aim to ensure all viewpoints are considered.

The Comprehensive ITP Program at BCDI, which is now in its tenth year, has received overwhelmingly positive feedback with regard to the active utilization of shared decision‐making and the focus on patient education and good health literacy.

A summary of the authors' guidance and recommendations for shared decision‐making in ITP is provided in Table 3.

**TABLE 3:** Guidance and recommendations for implementing and improving shared decision‐making in ITP.

Guidance for the implementation of shared decision‐making in ITP
Shared decision‐making should be incorporated for all ITP treatment decisions Both patients and caregivers should be involved in shared decision‐making, particularly in cases of pediatric ITP Patients should be educated on important aspects of treatment efficacy/effects, side effects, and medication burden so they can make informed decisions Trust between patients, their families and the healthcare team is essential Physicians should be trained in effective patient communication and shared decision‐making strategies
Recommendations for improved shared decision‐making in ITP
Develop shared decision‐making models specific to ITP Provide physician education to facilitate improved shared decision‐making: – Shared decision‐making approaches – Clear communication with patients – Assessment of patient health literacy – How to effectively assess patient goals and tailor treatment plans to meet those goals Develop tools to aid physicians in effective communication with patients Implementation of Comprehensive Care programs for patients with ITP

Abbreviation: ITP, immune thrombocytopenia.

## 7 Conclusions

Shared decision‐making in ITP, although a logical concept, is inconsistently applied. Developing models for shared decision‐making specific to ITP may improve the consistency of implementation. Individual patient factors and treatment priorities/concerns should always be considered with shared decision‐making, and the approach should be tailored to each individual patient. Implementation of Comprehensive Care programs for patients with ITP would likely improve overall patient care and provide an opportunity to actively utilize shared decision‐making.

## Author Contributions

**Shivi Jain:** conceptualization, writing – original draft, writing – review and editing. **Kelsey Kuhel:** writing – original draft, writing – review and editing. **Rose Cree:** writing – original draft, writing – review and editing. **Michael D. Tarantino:** conceptualization, writing – original draft, writing – review and editing.

## Disclosure

Michael D. Tarantino affirms that this manuscript is an honest, accurate, and transparent account of the study being reported; that no important aspects of the study have been omitted; and that any discrepancies from the study as planned (and, if relevant, registered) have been explained.

## Conflicts of Interest

The authors declare the following financial interests/personal relationships which may be considered as potential competing interests: Shivi Jain has served on advisory boards for Argenx and Global Blood Therapeutics Inc.; has served on speakers' bureaus for Novartis, Sanofi, and Global Blood Therapeutics Inc.; has participated as a speaker or director for continuing medical education courses for Clinical Viewpoints and PleXus Communication; and is a board member of the Sickle Cell Disease Association of Illinois. Kelsey Kuhel and Rose Cree declare no potential conflicts of interest with respect to the research, authorship, and/or publication of this article. Michael D. Tarantino is the Chief Medical and Chief Executive Officer of The Bleeding & Clotting Disorders Institute; is owner and president of Michael D. Tarantino, MD, SC, a private medical practice; serves on speakers' bureaus for Amgen, Grifols, Sanofi, and Sobi; and serves as a consultant for Amgen, Novartis, Sanofi, and Sobi.

## Data Availability

Data sharing is not applicable to this article, as no new data were created or analyzed in this study.
